# Reduction of Free Edge Peeling Stress of Laminated Composites Using Active Piezoelectric Layers

**DOI:** 10.1155/2014/439492

**Published:** 2014-06-16

**Authors:** Bin Huang, Heung Soo Kim

**Affiliations:** Department of Mechanical, Robotics and Energy Engineering, Dongguk University-Seoul, 30 Pildong-ro 1-gil, Jung-gu, Seoul 100-715, Republic of Korea

## Abstract

An analytical approach is proposed in the reduction of free edge peeling stresses of laminated composites using active piezoelectric layers. The approach is the extended Kantorovich method which is an iterative method. Multiterms of trial function are employed and governing equations are derived by taking the principle of complementary virtual work. The solutions are obtained by solving a generalized eigenvalue problem. By this approach, the stresses automatically satisfy not only the traction-free boundary conditions, but also the free edge boundary conditions. Through the iteration processes, the free edge stresses converge very quickly. It is found that the peeling stresses generated by mechanical loadings are significantly reduced by applying a proper electric field to the piezoelectric actuators.

## 1. Introduction

Laminated composite materials [1] have been increasingly attracted in the engineering industries, due to their high stiffness-to-weight ratio, high strength-to-weight ratio, high corrosion resistance, high chemical stability, and so on. They have been popularized in the aircraft manufacturing, vehicle industries, medical fields, and even sport equipment industries. With the improvement of manufacturing process and productivity, they have potential in replacing the conventional metal alloys and nonmetal materials massively in the future. However, it is well known that due to the mismatch of elastic properties between the adjacent layers, there exist large undesirable interlaminar peeling stresses. Because of these highly concentrated peeling stresses, failures such as delamination or crack may occur near the free edges and further lead to the damage of structures.

The concentrations of peeling stress often occur within a short distance from the free edges, known as the free edge boundary effect [2, 3]. The stresses decrease and vanish in the interior region of the laminates. Also, even an in-plane load applied on the laminate will cause 3D state of stress distributions near the free edge. Thus, some theories are not capable of predicting the free edge peeling stress distributions which assume the plane stress condition, such as classical laminate theory (CLT) [4, 5].

The concentrated free edge peeling stresses are critical to the fatigues of laminated structures. Some passive control methods have been developed either by adjusting the stacking sequence of laminates [6] or by changing the edge shape of fibers [7] to prevent the onset of delamination. Nevertheless, due to their passive nature, they cannot work effectively and other desired goals cannot be achieved. Thus, in this study, we will investigate the peeling stress reduction by using piezoelectric actuators. Owing to their electromechanical coupling nature, piezoelectric actuators have potential to generate stresses which have opposite signs of those peeling stresses generated by mechanical loadings and, in return, to reduce the magnitudes of those peeling stresses.

Since the last decades, a large volume of literature sources has introduced the modeling of smart composite laminated plates and beams [8–12]. Chen and Qiao investigated the electromechanical behavior and fracture of piezoelectric bilayer beams [13–15]. They also investigated the interlaminar stresses of plated beam with adhesive layers [16]. They found that the adhesive layers can affect the local deformations and in return alleviate the stress concentrations. While predicting the interlaminar stresses of laminated structures, most approaches neglect the adhesive layer effect. Numerous approximate approaches, without considering the adhesive layer effect, have been developed since there is no method of obtaining exact 3D elastic solutions. The developed theories vary from the displacement field based approaches [17] to stress field based approaches [18, 19]. All of these theories have their own characteristics and specific applications.

For the displacement field based equivalent single layer theories, such as classical laminate theory, first order shear deformation theory [20, 21], and higher order shear deformation theory [22–26], these theories are efficient in predicting the global response of laminates in bending, buckling, and vibration analysis, while they are not capable of predicting the localized free edge stress concentrations. The stress components are usually calculated from the constitutive relation or the equilibrium equations. Thus, the free edge stress concentration at the layer interface cannot be revealed accurately. For the stress function based equivalent single layer theories [27, 28], the localized free edge stress concentrations can be accurately predicted, while, on the other hand, displacement information cannot be achieved. Moreover, prescribed boundary conditions are mandatory in those equivalent single layer theories either for the preassumed displacement trial functions or for the stress trial functions. The layerwise theories [29] are much more accurate in analyzing the stress concentration at the ply level. For the displacement based layerwise theories, they allow the in-plane displacement to vary continuously through the laminate thickness, while their derivatives with respect to the thickness coordinate may be discontinuous through the thickness, thereby allowing for the possibility of continuous transverse stresses at the ply interfaces. For the stress based layerwise theories [30, 31], the stress fields are assumed layerwisely and transverse stress continuity conditions have to be enforced at the ply interfaces. Both the displacement and stress based theories can accurately predict the stress concentration at the free edges. However, the layerwise theories are much more computationally expensive than the equivalent single layer theories.

Among the analytical methods, another approximate method named as the extended Kantorovich method [32, 33] can be referred to. The extended Kantorovich method is an iterative method for solving partial differential equations. Although the initial assumption of admissible functions influences the convergence time a lot, the solutions can be improved gradually by repeating solving the governing equations and applying boundary and continuity conditions during the iteration processes. The assumed admissible functions with prescribed boundary conditions are of priority and any types of boundary condition problems can be solved by using this approach. The interlaminar stresses at the straight free edges of laminates under extension loads were investigated by Cho and Yoon [34]. And later Cho and Kim [35] analyzed the free edge interlaminar stresses of composite laminates under extension, bending, twisting, and thermal loads by using the extended Kantorovich method. In their study, they preferred using the stress trial functions, and traction-free boundary conditions and free edge boundary conditions were satisfied automatically during the iterative processes. Ungbhakorn and Singhatanadgid [36] proposed an extended Kantorovich method to investigate the buckling problem of rectangular laminates with various boundary conditions. The initial trial functions were used arbitrarily and the principle of minimum total potential energy was used to derive governing equations. Andakhshideh and Tahani [37] investigated the interlaminar stresses near free edges of finite length general composite laminates subjected to axial and shearing loads using three-dimensional extended Kantorovich method. They provided convergent 3D elasticity solutions with four terms of trial functions. Due to their arbitrary choice of initial trial functions and various boundary conditions, the extended Kantorovich method could be a good methodology in the interlaminar stress analysis of laminated composite structures.

In the present study, a stress function based extended Kantorovich method is proposed to investigate the reduction of free edge peeling stresses using piezoelectric actuators. Symmetrically layered laminates will be taken into consideration as symmetric laminates are most commonly used to avoid coupling of bending and twisting modes. Most studies start from the initial assumption that the strain or stress is independent of the longitudinal coordinate, and this study also adopts this point. The three-dimensional stress solutions are obtained by iterative processes involving multiterms of initially assumed trial functions. By the extended Kantorovich method, the influence of initial assumptions can be well eliminated and the accuracy of stress distributions can be improved a lot. Finally, convergent results are given as numerical examples and compared with those obtained by finite element method. It is expected to obtain significant peeling stress reduction by applying proper electric fields to piezoelectric actuators.

## 2. Mathematical Modeling

### 2.1. Derivation of Governing Equation

**Figure 1:**
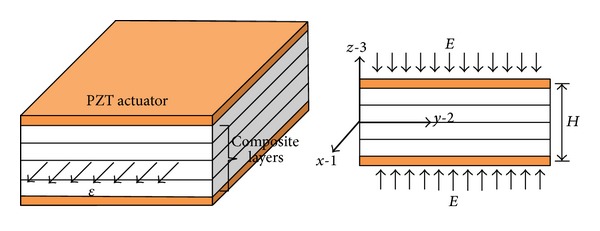
Geometry of laminated composite plate with surface bonded piezoelectric actuators under combined electromechanical loadings.

For the given geometry of laminated composite plate, as shown in Figure 1, two piezoelectric actuators are symmetrically surface bonded on the top and bottom surfaces of the laminates without considering the adhesive layer affect. This kind of structure is usually considered to be smart composite laminates [38]. Two piezoelectric layers are used to control the free edge peeling stresses that are generated by mechanical loadings. The longitudinal dimension of the laminates is considered long enough so that the stress fields are independent of the *x*-axis. Therefore, generalized plane strain states are assumed to be in the *x* direction.

The extended Kantorovich method requires iterative calculation methodology. By preassumption of trial functions and solving a set of ordinary differential equations, the solution of the first process can be obtained. Due to the rough initial assumption, it cannot guarantee the accuracy of solution. Thus, to overcome this weak point, iterative processes can be conducted to improve the accuracy of solution. The governing equations can be derived by using either Galerkin's method or the principle of virtual work. In this work, the principle of complementary virtual work is employed. The complementary strain energy of the laminated composite plate is calculated by the following equation:

(1) δU=∬sεiδσidA =∬sε2δσ2+ε3δσ3+ε4δσ4+ε5δσ5+ε6δσ6dA=0.

Before substituting strain and stress components into the complementary strain energy equation, an initially assumed stress field should be introduced. Instead of using displacement assumption, stress field assumption is preferred. A good choice for stress field is Lekhnitskii stress functions [39] which can express the three-dimensional stress state of piezo-bonded composite laminates. Lekhnitskii stress functions can automatically satisfy the pointwise equilibrium equations as follows:

(2) σ2=∂2F∂η2,  σ3=∂2F∂ξ2,  σ4=−∂2F∂ξ∂ησ5=−∂ψ∂ξ,  σ6=∂ψ∂η,

where *η* = *z*/*H* and *ξ* = *y*/*H* are nondimensionalized coordinates and *H* is the total thickness of the laminates. The solutions *F* and *ψ* are series combinations and assumed to be a combination of in-plane stress functions (*f*_*i*_(*ξ*) and *p*_*i*_(*ξ*)) and out-of-plane stress functions (*g*_*i*_(*η*)) using separation of variables as follows:

(3) $$\begin{array}{c} F=\overset{n}{\underset{i=1}{\sum }}f_{i}\left(\xi \right)g_{i}\left(\eta \right),\;\;\psi =\overset{n}{\underset{i=1}{\sum }}p_{i}\left(\xi \right)g_{i}^{\text{I}}\left(\eta \right). \end{array}$$

With the initial assumption of stress field, the strain tensor {*ε*_*i*_} can be found from the linear elastic constitutive relation. This relation is assumed for each individual layer with piezo induced strain term:

(4) $$\begin{array}{c} {\left\{\varepsilon_{i}\right\}}_{\left(k\right)}={\left[\overset{-}{S}\right]}_{\left(k\right)}{\left\{\sigma_{i}\right\}}_{\left(k\right)}+{\left[d\right]}_{\left(k\right)}{\left\{E\right\}}_{\left(k\right)},\;\left(i=1,2,\ldots ,6\right), \end{array}$$

where $\left[\overset{-}{S}\right]$ is the generalized compliance matrix for orthotropic materials. [*d*]  is the piezoelectric constant matrix and [*E*] is the applied electric field.

As the mechanical extension load is applied in the *x* direction, all other strain components can be expressed using *ε*_1_ and applied piezoelectric fields. Consider

(5) $$\begin{array}{c} \varepsilon_{i}={\hat{S}}_{ij}\sigma_{j}+\frac{S_{i1}}{S_{11}}\varepsilon_{1}+{\hat{d}}_{i3}E_{3},\;\left(i=2,3,\ldots ,6,\;\;j=2,3,\ldots ,6\right), \end{array}$$

where

(6) $$\begin{array}{c} {\hat{S}}_{ij}=S_{ij}-\frac{S_{i1}S_{1j}}{S_{11}},\;\;{\hat{d}}_{i3}=d_{i3}-\frac{S_{i1}}{S_{11}}d_{13}. \end{array}$$

Substituting (2) and (5) into (1), the complementary strain energy becomes

(7) δU=∬sεiδσidA =∬s[(S^ijσj+Si1S11ε1+d^3iE3)δσi]dξ dη=0,(i,j=2,3,…,6).

To simplify the above equation, integration by parts and free edge boundary conditions (*σ*_2_ = *σ*_4_ = *σ*_6_ = 0, at *ξ* = 0) are applied. Then, the double integration is simplified to the following equation and the governing equations can be obtained by using the variational principle. Consider

(8) ∫[aij(4)fjIV+aij(2)fjII+aij(0)fj+bij(2)pjII+bij(0)pj+ri]δfidξ +∫[dij(2)fjII+dij(0)fj+cij(2)pjII+cij(0)pj+si]δpidξ=0,  (i,j=1,2,…,n),

where

(9) aij(4)=∫S^33gigjdη,aij(2)=∫S^23(giIIgj+gigjII)dη−∫S^44giIgjIdη,aij(0)=∫S^22giIIgjIIdη,bij(2)=∫S^36gigjIIdη−∫S^45giIgjIdη, bij(0)=∫S^26giIIgjIIdη,cij(2)=−∫S^55giIgjIdη, cij(0)=∫S^66giIIgjIIdη,dij(2)=∫S^36giIIgjdη−∫S^45giIgjIdη,  dij(0)=∫S^26giIIgjIIdη,ri=∫(S21S11ε1+d^23E3)giIIdη,si=∫(S61S11ε1+d^63E3)giIIdη.

### 2.2. Solutions of Governing Equations and Generalized Eigenvalue Problem

To solve the fourth-order and second-order coupled ordinary differential equations, the trial functions *g*_*i*_(*η*)  are specified first in (10). Although prescribed boundary conditions are not necessarily satisfied for trial functions when using the extended Kantorovich method, the mode shapes of the clamped-clamped beam are used as the initial out-of-plane trail functions to satisfy the traction-free boundary conditions automatically (*σ*_3_ = *σ*_4_ = *σ*_5_ = 0  at  *η* = ±1/2). Consider

(10) $$\begin{array}{c} g_{i}\left(\eta \right)=\cos\left(\beta_{i}\eta \right)+\frac{\cos\left(\beta_{i}/2\right)}{\cosh\left(\beta_{i}/2\right)}\cosh\left(\beta_{i}\eta \right), \end{array}$$

where *β*_*i*_ are the roots of the following characteristic equation. Consider

(11) $$\begin{array}{c} \tan\left(\frac{\beta }{2}\right)+\tanh\left(\frac{\beta }{2}\right)=0. \end{array}$$

Thus, all coefficients in the governing equations can be obtained and the general solutions of governing equations can be assumed as the sum of homogeneous solutions (*f*_*i*_^(*H*)^  and  *p*_*i*_^(*H*)^) and particular solutions (*f*_*i*_^(*P*)^  and  *p*_*i*_^(*P*)^). The homogeneous solutions of *f*_*i*_^(*H*)^ and *p*_*i*_^(*H*)^ are assumed to be the following form:

(12) $$\begin{array}{c} f_{i}^{\left(H\right)}=v_{i}^{f}e^{\lambda \xi },\;\;p_{i}^{\left(H\right)}=v_{i}^{p}e^{\lambda \xi }. \end{array}$$

Substituting (12) into the governing equations and defining *v*_*j*_^*f*^II^^ = *λ* ^2^ *v*_*j*_^*f*^, the governing equations can be rewritten in the matrix form and a standard eigenvalue problem is formed. Consider

(13) [0−aij(4)−bij(2)00cij(0)I00]−1[aij(0)aij(2)bij(0)dij(0)dij(2)bij(0)0I0][vjfvjfIIvjp] =λ2[vjfvjfIIvjp], (i,j=1,2,…,n),

where *λ* ^2^ are eigenvalues of the above equation. Since the interlaminar stresses decay in the interior region of piezo-bonded composite laminates, only negative roots of *λ* ^2^ are selected. Equation (13) results in 3*n* eigenvalues and eigenvectors, so that the homogeneous solutions consist of 3*n* terms linear combination as follows:

(14) fi(H)=tjvijfe−λjξ,pi(H)=tjvijpe−λjξ, (i=1,2,…,n),  (j=1,2,…,3n),

where *t*_*j*_  are constants that will be determined by the prescribed boundary conditions.

The particular solutions are obtained by considering *f*_*i*_^(*P*)^ and *p*_*i*_^(*P*)^ as constants; thus all their derivatives are zeros in the governing equations and the remaining parts can be rewritten in the following equation:

(15) $$\begin{array}{c} \left[\begin{array}{c} f_{j}^{\left(P\right)}\\ p_{j}^{\left(P\right)} \end{array}\right]=-{\left[\begin{array}{cc} a_{ij}^{\left(0\right)} & b_{ij}^{\left(0\right)}\\ b_{ij}^{\left(0\right)} & c_{ij}^{\left(0\right)} \end{array}\right]}^{-1}\left[\begin{array}{c} r_{i}\\ s_{i} \end{array}\right]\;\left(i,j=1,2,\ldots ,n\right). \end{array}$$

The final step of solving the governing equations is to obtain the constants *t*_*j*_ in the homogeneous solutions. They can be calculated by enforcing the free edge boundary conditions (*σ*_2_ = *σ*_4_ = *σ*_6_ = 0  at  *ξ* = 0), and this will lead *f*_*i*_,  *f*_*i*_^I^, and *p*_*i*_ to be zeros at the free edges. Consider

(16) tjvijfe−λjξ|ξ=0+fi(P)=0,−tjλjvijfe−λjξ|ξ=0=0,tjvijpe−λjξ|ξ=0+pi(P)=0.

After solving the governing equations, all stress components can be obtained by substituting the solutions into the Lekhnitskii stress functions and they have the following expressions:

(17) σ2=∑i=1n ∑j=13n(tjvijfe−λjξ+fi(P))giII,σ3=∑i=1n ∑j=13nλi2tjvijfe−λjξgi,σ4=∑i=1n ∑j=13nλjtjvijfe−λjξgiI, σ5=∑i=1n∑j=13nλjtjvijpe−λjξgiI,σ6=∑i=1n ∑j=13n(tjvijpe−λjξ+pi(P))giII.

### 2.3. Iterative Procedures

The accuracy of the first step largely depends on the initial assumption of trail functions and the number of series terms involved in the trail functions. The results may also be influenced by the choice of trail functions which may cause singularity problem, harmonic distributions of stresses, and so forth. Thus, it is important to overcome the weaknesses appeared in the first process. We prefer using the extended Kantorovich method which is an iterative process. Through the iterative processes, the influence of the initial assumption can be eliminated and the convergent results can be obtained.

In the second step, the solutions of the first step (*f*_*i*_(*ξ*) and *p*_*i*_(*ξ*)) are considered to be the known functions and the principle of complementary virtual work is used again to derive governing equations. Here, the unknown out-of-plane stress functions (*g*_*i*_(*η*)) are assumed to be layer dependent and the continuity conditions are enforced at the layer interface. Thus, from the principle of complementary virtual work in (7) and integration by parts, the governing equation for the out-of-plane stress functions can be obtained as follows:

(18) ∫[mij(4)(k)gj(k)(IV)+mij(2)(k)gj(k)(II)+mij(0)(k)gj(k)+xi(k)] ×δgj(k)dη+Λ=0 (i,j=1,2,…,n),(k=1,2,…,N),

where Λ represents the boundary terms induced by integration by parts and the superscript (*k*) refers to the *k*th ply. The layerwise coefficients are listed as follows:

(19) mij(4)(k)=∫[S^22(k)fifj+S^26(k)(fipj+pifj)+S^66(k)pipj]dξ,mij(2)(k)=∫[S^23(k)(fifjII+fiIIfj)+S^36(k)(fiIIpj+pifjII) −S^44(k)fiIfjI−S^45(k)(fiIpjI+piIfjI)−S^55(k)piIpjI]dξ,mij(0)(k)=∫S^33(k)fiIIfjIIdξ,   xi(k)=∫(S13(k)S11(k)ε1+d^33E3)fiIIdξ.

The solution procedure is the same as in the first step. A generalized eigenvalue problem is solved and the general solution is calculated by adding the homogeneous solutions *g*_*i*_^(*k*)(*H*)^ and the particular solutions *g*_*i*_^(*k*)(*P*)^:

(20)  gi(k)=bj(k)vig(k)eμ(k)η+gi(k)(P),(k=1,2,…,N), (i=1,2,…,n),

where *μ* ^(*k*)^ are the roots of eigenvalues and *v*_*i*_^*g*(*k*)^ are the eigenvectors.

The constants *b*_*j*_^(*k*)^ in the homogeneous solutions can be determined from the traction-free boundary conditions on the top and bottom surfaces (*σ*_*i*_ = 0  at  *η* = ±1/2, *i* = 3,4, 5), interlaminar stress continuity conditions at the ply interfaces (*σ*_*i*_^+^(*k*) = *σ*_*i*_^−^(*k* + 1), *i* = 3,4, 5), and boundary terms Λ set to be zero. These conditions lead to the following equations:

(21) gi(k)=bj(k)vig(k)eμ(k)η|η=±1/2+gi(k)(P)=0, (k=1,N),gi(k)I=bj(k)μ(k)vig(k)eμ(k)η|η=±1/2=0, (k=1,N),bj(k)vig(k)eμ(k)η|η=ηk++gi(k)(P)=bj(k+1)vig(k+1)eμ(k+1)η|η=ηk+1− +gi(k+1)(P), (k=1,2,…,N−1),bj(k)μ(k)vig(k)eμ(k)η|η=ηk+=bj(k+1)μ(k+1)vig(k+1)eμ(k+1)η|η=ηk+1−, (k=1,2,…,N−1).

Thus, after obtaining the solutions of governing equation, all stress components can be obtained and are listed as follows:

(22) σ2=∑i=1n ∑j=14nfi(ξ)[μj(k)2vijg(k)bj(k)eμj(k)η],σ3=∑i=1n ∑j=14nfiII(ξ)[vijg(k)bj(k)eμj(k)η+gi(k)(P)],σ4=∑i=1n ∑j=14n−fiI(ξ)[μj(k)νijg(k)bj(k)eμj(k)η],σ5=∑i=1n ∑j=14n−piI(ξ)[μj(k)νijg(k)bj(k)eμj(k)η],σ6=∑i=1n ∑j=14npi(ξ)[μj(k)2νijg(k)bj(k)eμj(k)η].

After conducting the second step, if the results are still not convergent, more processes can be conducted. The iterative algorithm can be concluded as follows.

1. Assume an initial set of out-of-plane stress functions (*g*_*i*_(*η*)) and then take the principle of complementary virtual work to solve the in-plane stress functions (*f*_*i*_(*ξ*) and *p*_*i*_(*ξ*)).
2. Use the in-plane stress functions (*f*_*i*_(*ξ*) and *p*_*i*_(*ξ*)) obtained from the first step as the known functions and repeat taking the principle of complementary virtual work to solve the out-of-plane stress functions (*g*_*i*_(*η*)).
3. Use the out-of-plane stress functions (*g*_*i*_(*η*)) obtained from the second step as the known functions and repeat taking the principle of complementary virtual work to solve the in-plane stress functions (*f*_*i*_(*ξ*) and *p*_*i*_(*ξ*)).

Three steps are narrated in the above algorithm and more processes can be conducted, if required. However, one essential difference should be emphasized. In the first step, the initially assumed out-of-plane stress functions represent the global state of stress distributions which implies that the first step is a stress based equivalent single layer theory, while from the second step, the out-of-plane stress functions are assumed layerwisely and continuity conditions should be enforced at the layer interfaces. All stress components satisfy the boundary conditions automatically when using the extended Kantorovich method.

## 3. Numerical Results

Based on the theoretical formulations discussed in the previous section, numerical examples are investigated. The laminated composite structures are subjected to uniaxial extension and electric load. The material properties of graphite/epoxy composite laminates [40] and piezoelectric actuator (PZT-5H) are given in Table 1. The lamina thickness is 0.125 mm, which is almost one-fourth of the PZT thickness (0.5 mm). For convenience, 4 laminae, which have the same fiber orientations, are considered one lamina, so that each lamina thickness is then the same as the PZT thickness. The width of the laminate *b* is equal to four times the total thickness *H*.

### 3.1. Convergence Study

The laminates are subjected to uniaxial tension only in this part. To investigate the convergence of the proposed method, different terms of initially assumed stress functions are used and several iterative processes are conducted. To validate the proposed modeling, 3D finite element analysis is also conducted using commercial software ANSYS. The Solid 64 element is used for composite laminates and Solid 5 is used for PZTs. To obtain accurate interlaminar stress distributions, fine mesh is required and it should be refined at the layer interface. Thus, we used 20 elements along the thickness direction for a single layer. The total degree of freedom is 640,281 that is much larger than our proposed method. The three-dimensional solid laminate has a relatively long length, compared with the width and height, to express the generalized plane strain state effectively.

**Figure 2:**
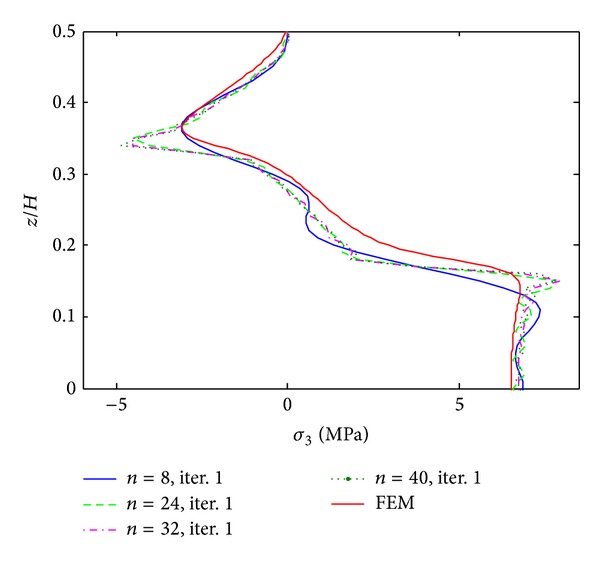
*σ*_3_ distribution of [PZT/0_4_/90_4_]s laminate at the free edge, using different terms, under 0.1% uniaxial strain.

Figure 2 shows interlaminar normal stress (*σ*_3_) distribution through the thickness direction at the free edge of [PZT/0_4_/90_4_]s laminate under 0.1% uniaxial strain obtained from the first process. The result is compared with the FEM result, and the influence of term number used in the first process is investigated. It is found that the accuracy of the stress distribution largely depends on the number of terms in the first process. With 8 terms of initially assumed stress functions, the result cannot converge well and undesired oscillation appears due to the initial assumption of harmonic functions. Moreover, the stress concentration at the layer interfaces also cannot be predicted accurately. As we expected the peak values should be exactly located at the PZT/0 layer interface (*z*/*H* = 1/3) and 0/90 layer interface (*z*/*H* = 1/6), while it cannot be recovered by using 8 terms. Only by increasing the number of terms, the locations can be predicted more precisely. Until using 40 terms of initially assumed stress functions, the stress concentrations can be found exactly located at the layer interfaces, but undesired oscillations still exist. The proposed result can be verified by the FEM result. Both of them show the same tendency and magnitude, whereas the result obtained by finite element method shows difficulties in predicting the stress concentrations at the layer interfaces even using fine mesh. A proper choice of the term number not only guarantees the accuracy of results, but also improves the computational ability. It is to be noted that the computational time using 40 terms for solving the first process only costs seconds, while FEM takes 10–20 minutes to run a single case.

**Figure 3:**
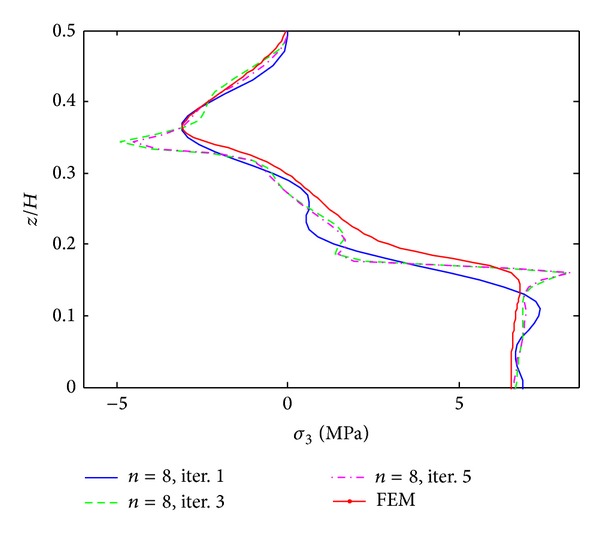
*σ*_3_ distribution of [PZT/0_4_/90_4_]s laminate at the free edge from different iterations, under 0.1% uniaxial strain.

**Figure 4:**
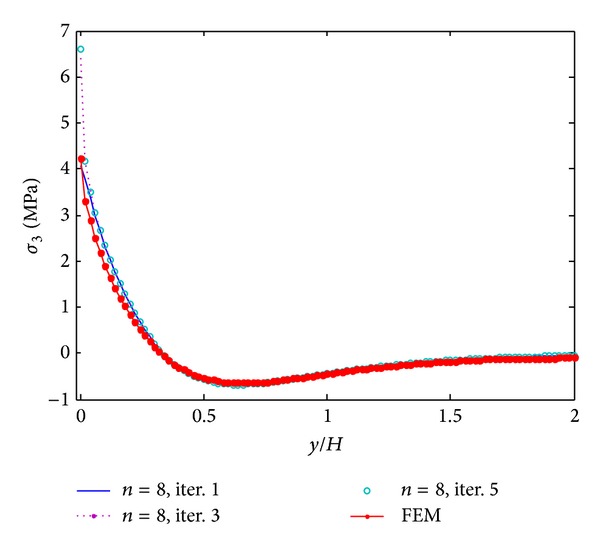
*σ*_3_ distribution of [PZT/0_4_/90_4_]s laminate at the interface of *z*/*H* = 1/6 from different iterations, under 0.1% uniaxial strain.

**Figure 5:**
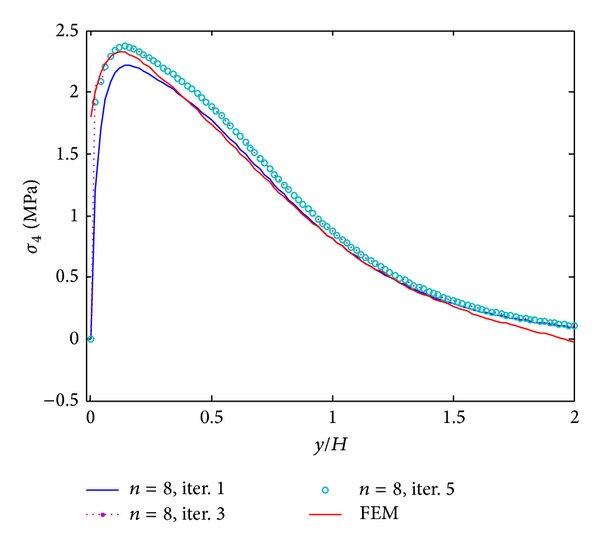
*σ*_4_ distribution of [PZT/0_4_/90_4_]s laminate at the interface of *z*/*H* = 1/6 from different iterations, under 0.1% uniaxial strain.

The results of iterative process for [PZT/0_4_/90_4_]s laminates under 0.1% uniaxial strain are given in Figures 3–5. These results are obtained by only 8 terms and different iterative processes.  Figure 3 shows the interlaminar normal stress (*σ*_3_) distribution through the thickness direction at the free edge. From the graph, it is found that the iterative processes can significantly improve the accuracy of the stress prediction. The locations of stress concentration can be exactly predicted at the layer interfaces and the oscillations appeared in the first iteration disappeared since the third iteration. Figures 4 and 5 show interlaminar normal stress distribution (*σ*_3_) and interlaminar shear stress distribution (*σ*_4_) through the in-plane direction, respectively. First iteration with 8 terms of initially assumed stress functions cannot guarantee the convergence of the stresses, while the third and fifth iterations provide almost converged stresses. The stresses obtained by FEM show great coincidence with those obtained by the proposed method but finite element analysis underestimates stress concentrations at the free edge. Interlaminar shear stress (*σ*_4_) also cannot satisfy the free edge boundary condition exactly in finite element analysis. It is concluded that the iterative processes can provide the converged stresses with small number of terms. Not only the influence of initial assumption can be eliminated, but also the stress concentration locations can be predicted accurately by the iterative processes. In the following work, the results of the fifth iteration with 8 terms of initially assumed that the stress functions are considered to investigate the reduction of peeling stresses by piezoelectric excitation.

### 3.2. Reduction of Peeling Stresses Using Piezoelectric Excitations

The existence of peeling stresses generated by the mechanical loadings can be reduced by applying proper electric fields to the actuators. Due to the electromechanical coupling phenomenon, the induced strain or stress generated by the electric loadings has the possibility of compensating the strain or stress generated by the mechanical loadings, in turn, to reduce the peeling stresses and to expand the service life of laminated structures. To investigate the feasibility of peeling stress reduction using piezoelectric actuators, two general lay-ups are considered and numerical examples are discussed in this part.

**Figure 6:**
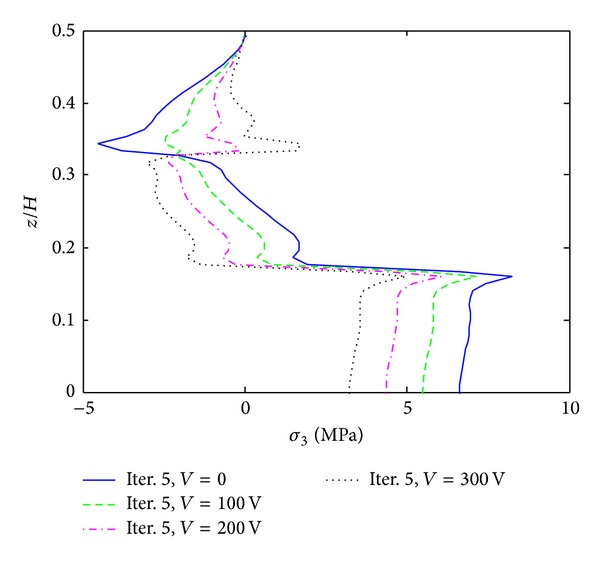
*σ*_3_ distribution of [PZT/0_4_/90_4_]s laminate at the free edge, under 0.1% uniaxial strain.

**Figure 7:**
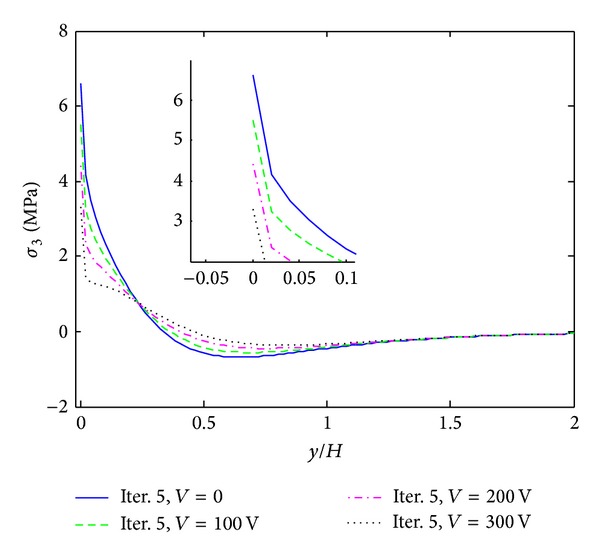
*σ*_3_ distribution of [PZT/0_4_/90_4_]s laminate at the interface of *z*/*H* = 1/6, under 0.1% uniaxial strain.

**Figure 8:**
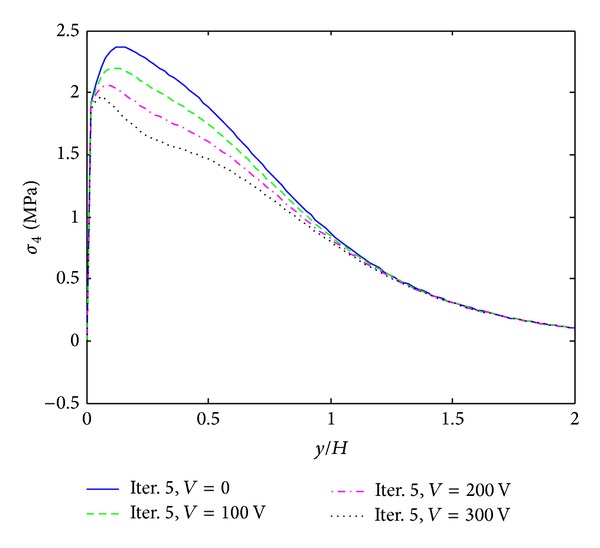
*σ*_4_ distribution of [PZT/0_4_/90_4_]s laminate at the interface of *z*/*H* = 1/6, under 0.1% uniaxial strain.

The first example is given by cross ply composite laminate ([PZT/0_4_/90_4_]s) which is subjected to 0.1% uniaxial strain. Moreover, different electric voltages are applied to the actuators that are expected to reduce the free edge peeling stresses.  Figure 6 shows interlaminar normal stress (*σ*_3_) distribution through the thickness direction at the free edge. It can be clearly found that the peeling magnitude of *σ*_3_ is significantly reduced at the 0/90 layer interface by applying a positive electric voltage to the actuators. Without electric potential, the concentrated peeling stress is 8.3 MPa. It is reduced to 4.9 MPa, when 300 V electric potential is applied. This is of great meaningfulness to the reduction of positive peeling stress at the interface. Figures 7 and 8 show interlaminar normal stress (*σ*_3_) and interlaminar shear stress (*σ*_4_) distribution through the in-plane direction at the 0/90 interface, where *z*/*H* = 1/6. Our ambition is to reduce the peeling stress at the composite layer interface by using piezoelectric actuator. Thus, this interface is what we concerned. *σ*_3_ and *σ*_4_ show large positive values at the free edge and near the free edge under the mechanical loading. With the increasing of the electric potential, the magnitudes of peeling stresses are reduced dramatically. The peeling stress *σ*_3_ can be reduced to almost 50.2 percent when 300 V electric potential is applied which is summarized in Table 2. For interlaminar shear stress (*σ*_4_), 17.3 percent decrement is observed when 300 V electric potential is applied.

**Figure 9:**
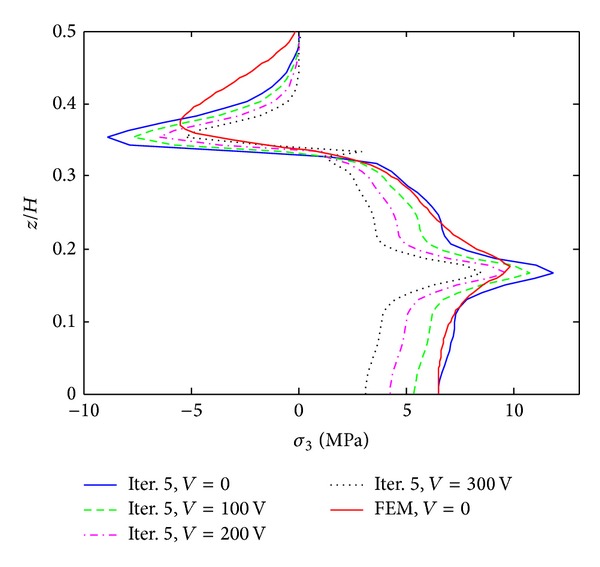
*σ*_3_ distribution of [PZT/45_4_/−45_4_]s laminate at the free edge, under −0.1% uniaxial strain.

**Figure 10:**
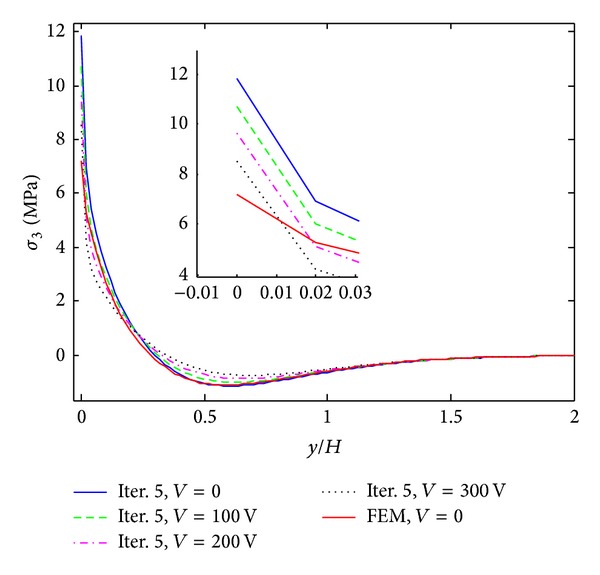
*σ*_3_ distribution of [PZT/45_4_/−45_4_]s laminate at the interface of *z*/*H* = 1/6, under −0.1% uniaxial strain.

**Figure 11:**
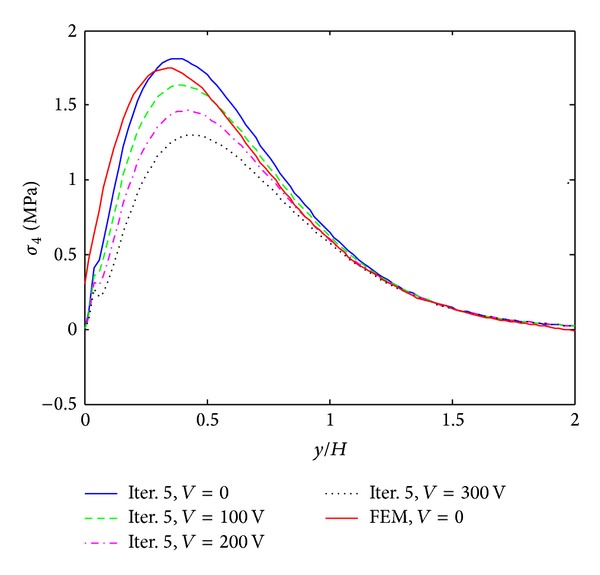
*σ*_4_ distribution of [PZT/45_4_/−45_4_]s laminate at the interface of *z*/*H* = 1/6, under −0.1% uniaxial strain.

**Figure 12:**
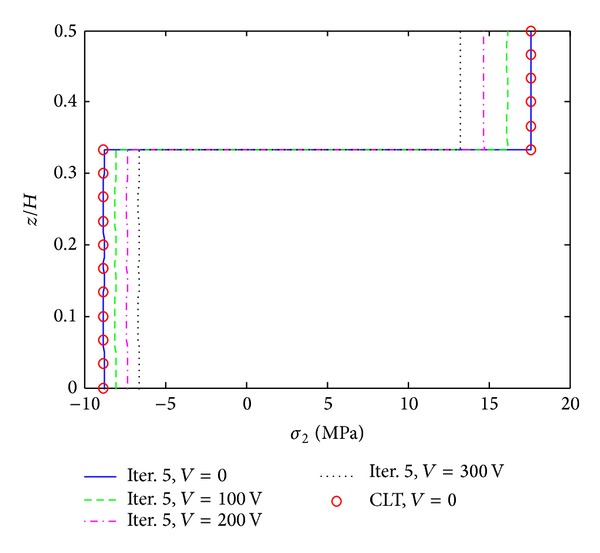
*σ*_2_ distribution of [PZT/45_4_/−45_4_]s laminate at the interior, under −0.1% uniaxial strain.

The second example is given by angle ply composite laminate ([PZT/45_4_/−45_4_]s) which is subjected to −0.1% uniaxial strain load. The laminate is subjected to compressive load while it sustains large peeling stresses at the 45/−45 layer interfaces.  Figure 9 shows interlaminar normal stress (*σ*_3_) distribution through the thickness direction at the free edge. It is found that the peeling stress is severely concentrated at the 45/−45 layer interface which could lead to the onset of delamination of composite layers. However, this peeling stress can also be significantly reduced in angle ply laminate, when a positive electric potential is applied to the actuators. The peak value is reduced from 11.83 MPa to 8.52 MPa. The interlaminar normal stress (*σ*_3_) and interlaminar shear stress (*σ*_4_) distribution through the in-plane direction at the 45/−45 layer interface are given in Figures 10 and 11, respectively. The interlaminar normal stress (*σ*_3_) and interlaminar shear stress (*σ*_4_) well match with those obtained by the finite element analysis for the mechanical loading case. However, finite element analysis still underestimates the stress concentration at the free edge. The detailed reductions of peeling stresses can be found in Table 3. Since angle ply composite laminates sustain larger shear stresses than cross ply composite laminates, the reduction of *σ*_3_ is smaller than that of cross ply, while the reduction of *σ*_4_ is larger than that of the cross ply. The proposed reduction algorithm also works for the in-plane stress (*σ*_2_) which is obtained at the interior of the laminate, shown in Figure 12. It is interesting to find that the magnitude of *σ*_2_ also decreases when a positive electric potential is applied. This result can be verified by using classical laminate theory (CLT). The fifth result can accurately recover the CLT solution. Thus, the proposed reduction approach is well suited to the angle ply composite laminate.

## 4. Conclusions

In this study, the reduction of free edge peeling stresses of laminated composite structures using piezoelectric actuators was analyzed by using the extended Kantorovich method. Multiterms of the initially assumed stress functions were involved, and three-dimensional stress distributions were obtained. The accuracy and effectiveness of the present approach were demonstrated by comparing the stresses obtained by ANSYS. The provided results showed excellent agreements with those obtained by FEM, while it showed accuracy and great efficiency in computation. It was found that small terms of trail functions could lead to accurate solutions. The present method is general and applicable to various lay-up configurations. The peeling stresses generated by mechanical load at the free edges were significantly reduced by applying a proper electric potential. The present method could serve as an efficient tool for reducing the free edge peeling stresses and extending the service life of laminated composite structures.

## Figures and Tables

**Table 1:** Material properties of composite lamina and PZT-5H actuator.

Composite	Actuator
*E*_1_ = 138 GPa	*E*_1_ = *E*_2_ = 62 GPa
*E*_2_ = *E*_3_ = 14.5 GPa	*E*_3_ = 48 GPa
*G*_12_ = *G*_13_ = *G*_23_ = 5.9 GPa	*G*_12_ = 23.5 GPa, *G*_13_ = *G*_23_ = 23.5 GPa
*ν*_12_ = *ν*_23_ = *ν*_21_ = 0.21	*ν*_12_ = *ν*_23_ = *ν*_21_ = 0.29
	*d*_31_ = *d*_32_ = 274 × 10^−12^ m/V
	*d*_33_ = −593 × 10^−12^ m/V, *d*_15_ = −741 × 10^−12^ m/V

**Table 2:** Comparison of peak stresses for cross ply composite laminate ([PZT/0_4_/90_4_]s) at the 0/90 layer interface, where *z*/*H* = 1/6, under 0.1% uniaxial strain, fifth iteration, *n* = 8.

Applied electric potential (V)	*σ*_3_		*σ*_4_	
	(MPa)	Reduction (%)	(MPa)	Reduction (%)
0	6.62	0	2.37	0
100	5.51	16.8%	2.19	7.6%
200	4.41	33.4%	2.06	13.1%
300	3.30	50.2%	1.96	17.3%

**Table 3:** Comparison of peak stresses for angle ply laminate ([PZT/45_4_/−45_4_]s) at the 45/−45 layer interface, where *z*/*H* = 1/6, under −0.1% uniaxial strain, fifth iteration, *n* = 8.

Applied electric potential (V)	*σ*_3_		*σ*_4_	
	(MPa)	Reduction (%)	(MPa)	Reduction (%)
0	11.28	0	1.81	0
100	10.73	9.3%	1.63	10.0%
200	9.63	18.6%	1.46	19.3%
300	8.52	24.5%	1.30	28.2%
